# Serum albumin at 1 year after peritoneal dialysis predicts long-term outcomes on continuous ambulatory peritoneal dialysis

**DOI:** 10.1080/0886022X.2022.2033264

**Published:** 2022-02-15

**Authors:** Li Jin, Xiaopei Wang, Ying Ma, Jie Zheng, Wanhong Lu, Liyi Xie, Jing Lv

**Affiliations:** a Department of Nephrology, Kidney Hospital, The First Affiliated Hospital, School of Medicine, Xi'an Jiaotong University, Xi'an City, China; b Clinical Research Center, The First Affiliated Hospital, School of Medicine, Xi'an Jiaotong University, Xi'an City, China

**Keywords:** Peritoneal dialysis, serum albumin, mortality

## Abstract

**Background:**

Hypoalbuminemia at baseline is a powerful predictor of long-term outcomes in peritoneal dialysis patients. However, the levels of serum albumin are dynamically changed during PD. The present study investigated whether the improvement of hypoalbuminemia during PD can affect the patients’ outcomes.

**Methods:**

436 consecutive incidents continuous ambulatory peritoneal dialysis patients were involved in this study. Demographic, hematologic, biochemical, and dialysis-related data at baseline as well as 1 year after PD were collected. All patients were followed for at least 1 year for mortality.

**Results:**

Among the 436 patients, the mean age was 48.44 ± 14.98 years, with 58.26% males and 18.12% prevalence of diabetes. The mean follow-up time was 48.25 ± 24.05 months. During the follow-up period, a total of 68 patients died. Serum albumin was 34.35 ± 5.65 g/L at baseline, which increased to 37.39 ± 5.05 g/L at 1 year after PD. Multivariate linear regression analysis showed that sex, age, BMI, diabetic nephropathy, as well as albumin at baseline were independently associated with albumin at 1 year. Every 1 year of age rise would result in a 3.9% increase in the risk of mortality (HR = 1.039, 95%CI 1.016–1.061, *p* = 0.001). Every 1 g/L increase in albumin at 1 year after PD confers an 8.7% decrease in the risk of mortality (HR = 0.913, 95%CI 0.856–0.973, *p* = 0.005).

**Conclusion:**

The level of serum albumin was increased in the first year of PD. Serum albumin after 1 year of PD predicted mortality in peritoneal dialysis.

## Introduction

Peritoneal dialysis (PD) is an important renal replacement therapy for end-stage kidney disease (ESKD) patients. Previous studies have demonstrated that hypoalbuminemia at baseline is a powerful predictor of cardiovascular disease-related mortality as well as all-cause mortality [1–4]. However, serum albumin is influenced by many factors, such as nutritional status [5,6], inflammation [7,8], diabetes mellitus [9], urine and peritoneal protein loss [10], extracellular fluid volume [11], and the presence of comorbid disease [12]. The levels of serum albumin were dynamically changed according to different clinic conditions during peritoneal dialysis. We wonder whether the changes of serum albumin during PD can affect the patients’ outcomes.

In the past, it was reported that serum albumin achieved the peak levels after 1 year of PD, regardless of the initial albumin levels [13]. Moreover, after achieving the peak level of serum albumin, all the patients have similar albumin trajectories [13]. Therefore, in the present study, we analyzed the change of albumin during the first year of PD and further investigated whether the improvement of hypoalbuminemia during PD can affect the patients’ outcomes.

## Methods

### Design

**Figure 1.**
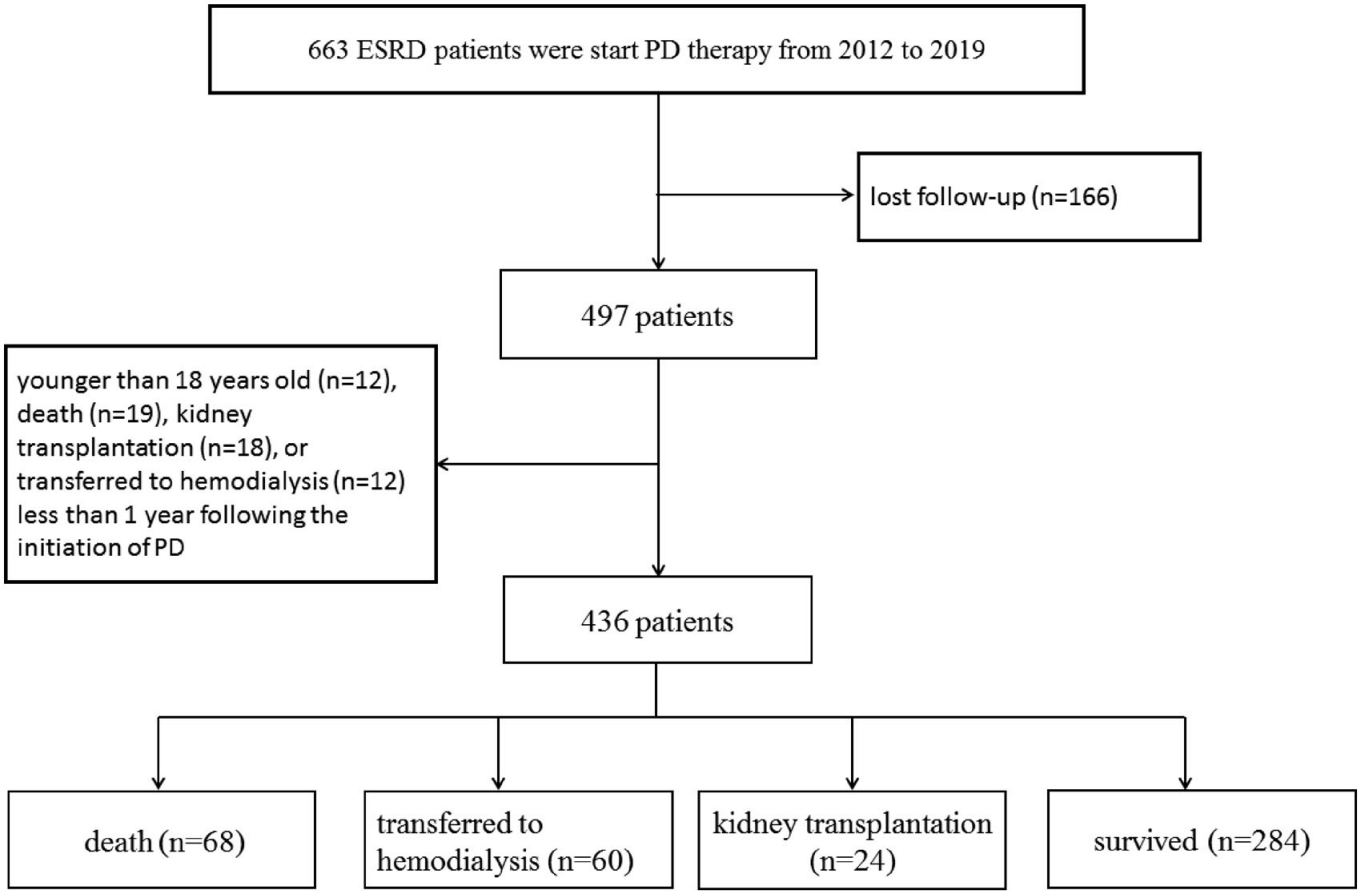
Flow chart of the participants in the study cohort. PD: peritoneal dialysis.

From January 2012 to January 2019, there were a total of 663 ESKD patients who start peritoneal dialysis therapy in the First Affiliated Hospital of Xi'an Jiaotong University. 227 patients were excluded for the following reasons: lost follow-up (*n* = 166), younger than 18 years old (*n* = 12), death (*n* = 19), kidney transplantation (*n* = 18), or transferred to hemodialysis (*n* = 12) less than 1 year following the initiation of PD (Figure 1). Finally, there were 436 CADP patients included in the study. All patients were followed up to May 2020. The long-term outcomes were obtained through outpatient review, letter, or telephone follow-up. Patient survival, time of death, cause of death, transfer to hemodialysis or kidney transplantation were recorded. The study was approved by the Clinical Research Ethics Committee of the First Affiliated Hospital of Xi'an Jiaotong University (application ID: XJTU1AF2019LSL-017).

### Data collection

Baseline demographic and clinical data including the cause of ESKD and laboratory measurements at the start of PD such as hemoglobin, albumin, Ca, P, PTH, and estimated glomerular filtration rate (eGFR) were recorded by chart review. All patients were given conventional glucose-based solution and used CAPD on initial PD. The peritoneal equilibrium test, baseline PD fluid, and urine protein loss as well as dialysis adequacy were performed around 4 weeks after starting dialysis. After 1 year of PD, clinical data as well as protein loss, dialysis adequacy were collected again. All patients were prescribed to achieve the target of weekly Kt/V urea >1.7.

### Statistical analysis

Statistical analysis was performed using SPSS software 13.0 (SPSS Inc., Chicago, IL). Continuous variables were summarized as means ± standard deviation and analyzed using Student’s *t*-test. The relationship between albumin and other variables was examined by the Pearson correlation coefficient. Multiple linear regression was performed to determine the independent associations of albumin, using variables identified in the univariate model. The Kaplan–Meier survival analysis was conducted to compare patient mortality. The Cox regression analysis was performed to analyze the risk factors, calculate the hazard ratio (HR), adjusted HR (AHR), and 95% confidence interval (CI). A *p*-value < 0.05 was considered statistically significant. All probabilities were two-tailed.

## Results

### Patient demographics

We studied 436 new CAPD patients, all of which received PD solution with low glucose degradation product. The mean age was 48.44 ± 14.98 years. Among them, 254 patients (58.26%) were male. The mean follow-up time was 48.25 ± 24.05 months. The demographic and baseline clinical characteristics are summarized in Tables 1 and 2. The major causes of ESKD were glomerulonephritis (*n* = 298, 68.35%) and diabetic nephropathy (*n* = 79, 18.12%).

**Table 1.** Demographics and biochemical characteristics.

	Baseline
No. of patients	436
Gender (M: F)	254:182
Age (years)	48.44 ± 14.98
BMI (kg/m^2^)	21.99 ± 3.49
Primary renal disease	
Glomerulonephritis	298 (68.35%)
Diabetic nephropathy	79 (18.12%)
Hypertensive nephrosclerosis	35 (8.02%)
Interstitial nephritis	14 (3.21%)
Lupus nephritis/Henoch–Schonlein purpura nephritis	4 (0.92%)
ANCA-associated vasculitis	4 (0.92%)
Others	2 (0.46%)
eGFR (mL/min1.73m^2^)	6.79 ± 3.23

*Note*: Baseline was defined as within 1 week before initiation of PD therapy.

### The changes of serum albumin

**Figure 2.**
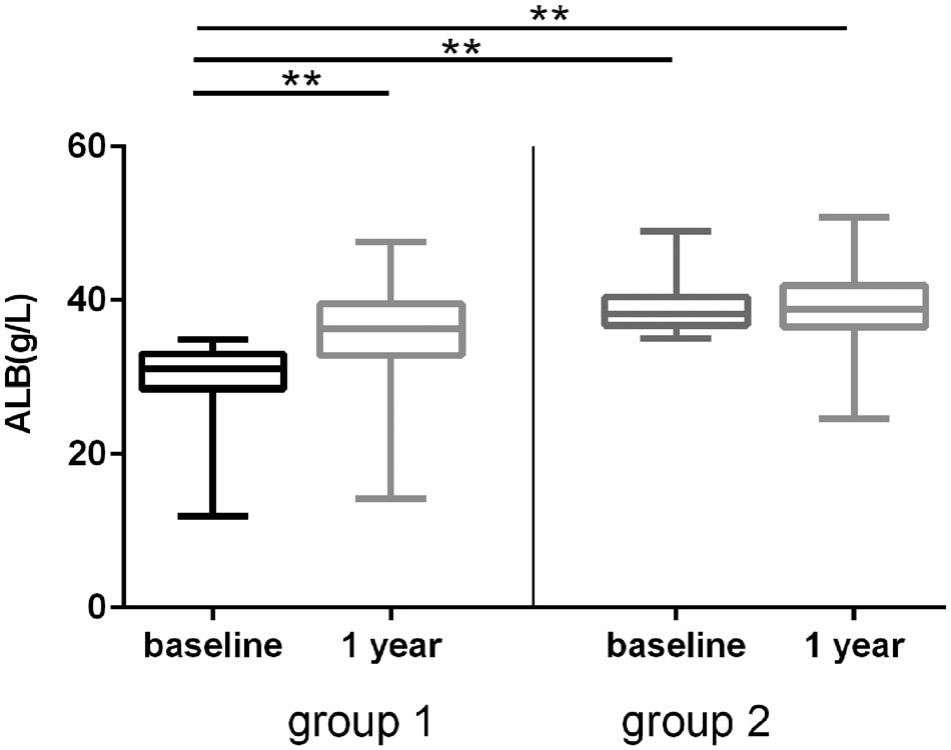
Albumin change at baseline and 1 year after PD therapy. The patients were divided into hypoproteinemia (group A, albumin <35 g/L) and non-hypoproteinemia (group B, albumin >35 g/L) according to the levels of serum at baseline. The changes of serum albumin at 1 year after PD were compared. Error bars denote standard deviations. **p < 0.001.

Next, we analyzed the change of serum albumin after 1 year of PD. Mean albumin was 34.35 ± 5.65g/L at baseline, which was increased to 37.39 ± 5.05g/L at 1 year after PD (Table 2). The low albumin (ALB < 35g/L) existed in 50.69% (221/436) patients at baseline, the proportion of which decreased to 27.98% (122/436) at 1 year after PD. In the 221 patients, whose albumin <35 g/L at baseline, the albumin raised from 30.04 ± 4.12g/L baseline to 35.80 ± 5.21g/L after 1 year of PD (*p* < 0.001). In the 215 patients, whose albumin ≥ 35g/L patients at baseline, the albumin increased from 38.81 ± 2.85 baseline to 39.03 ± 4.03 g/L at 1 year after PD (*p* = 0.474) (Figure 2).

**Table 2.** Peritoneal transport, dialysis adequacy, and biochemical characteristics at baseline and 1 year after PD therapy.

Variables (normal ranges)	Baseline	1 year	P value
BMI (kg/m^2^)	18.50 ± 3.62	22.48 ± 3.49	<0.001
Mean arterial pressure (mmHg)	119.60 ± 25.46	116.00 ± 18.1	0.013
Urea (2.60–7.50 mmol/L)	29.35 ± 11.79	18.21 ± 5.18	<0.001
Creatinine (41–73 μmol/L)	813.83 ± 301.55	789.78 ± 268.03	0.089
Albumin (40–55 g/L)	34.35 ± 5.65	37.39 ± 5.05	<0.001
Hemoglobin (115–150 g/L)	77.91 ± 27.10	105.24 ± 17.96	<0.001
Cholesterol (3.10–5.69 mmol/L)	4.14 ± 3.24	4.62 ± 1.59	0.004
Calcium (2.11–2.52 mmol/L)	1.95 ± 0.31	2.12 ± 0.21	<0.001
Phosphorus (0.85–1.51 mmol/L)	1.66 ± 0.72	1.41 ± 0.37	<0.001
Parathyroid hormone (15.0–65.0 pg/ml)	301.39 ± 274.72	375.04 ± 248.47	<0.001
D/P4 Cr	0.65 ± 0.14		
C-reaction protein (0–3 mg/L)	2.18 ± 3.34	3.69 ± 7.44	<0.001
Urine volume (mL)	1077.02 + 504.21	729.41 ± 498.96	<0.001
Total Kt/V urea	2.10 ± 0.62	2.05 ± 0.60	0.201
Total Ccr (L/weeks/1.73 m^2^)	90.94 ± 42.20	83.98 ± 48.34	0.006
nPCR	0.96 ± 0.82	0.96 ± 1.22	0.997
Protein loss			
Proteinuria (g/day)	1.16 ± 1.42	0.76 ± 1.27	<0.001
PD effluent protein loss (g/day)	4.16 ± 2.47	5.03 ± 2.61	<0.001

D/P4: dialysate-to-plasma creatinine ratio in 4 h; nPCR: normalized protein catabolic rate.

**Table 3.** biochemical characteristics associated with ALB at 1 year after PD therapy.

	Univariate^1^		Multivariate^2^	
	*r*	*p*	*Β*	*p*
Gender	−0.150	0.002	−0.152	0.001
Age	−0.279	0.000	−0.246	0.000
BMI (kg/m^2^)	0.154	0.001	0.133	0.005
Diabetic nephropathy	0.188	0.000	0.097	0.047
Baseline				
Urine (ml)	0.118	0.015	0.038	0.338
Albumin (g/L)	0.340	0.000	0.338	0.000
Hemoglobin (g/L)	0.119	0.013	0.057	0.196
PD effluent protein loss (g/day)	−0.109	0.026	−0.042	0.337

Data are compared by ^1^Pearson’s correlation coefficient; and ^2^multiple linear regression.

### Factors associated with albumin at 1 year of PD

Further, we analyzed the factors associated with albumin at 1 year after PD. We found that there were modest but statistically correlations between albumin at 1 year and sex, age, BMI, diabetic nephropathy, urine volume, albumin, hemoglobin, as well as PD effluent protein loss at baseline. Multivariate linear regression analysis showed that sex, age, BMI, diabetic nephropathy, as well as albumin at baseline were independently associated with albumin at 1 year after PD (Table 3).

### Survival

The mean follow-up time was 48.25 ± 24.05 (12.07–158.98) months. During the follow-up, 68 (15.60%) patients died, 60 patients were transferred to hemodialysis, 24 patients had kidney transplantation, 3 patients had recovery of renal function. The causes of death were cardiac disease (24 cases), infection (including peritonitis and non-peritonitis infection, 17 cases), cerebrovascular disease (14 cases), cancer (3 cases), gastrointestinal bleeding (3 cases), other unknown reasons (7 cases).

**Table 4.** Cox regression analysis of patient survival.

	Univariate		Multivariate	
	HR	*p* value	Adjusted HR	*p* value
Age	1.048	<0.001	1.039	0.001
Baseline				
Urine (mL)	0.999	0.017	0.999	0.096
1 year				
Albumin	0.904	<0.001	0.913	0.005
Prealbumin	0.997	0.018	1.000	0.812
Parathyroid hormone	0.997	0.001	1.018	0.159
C-reaction protein	1.026	0.008	0.999	0.135

HR: hazard ratio.

**Figure 3.**
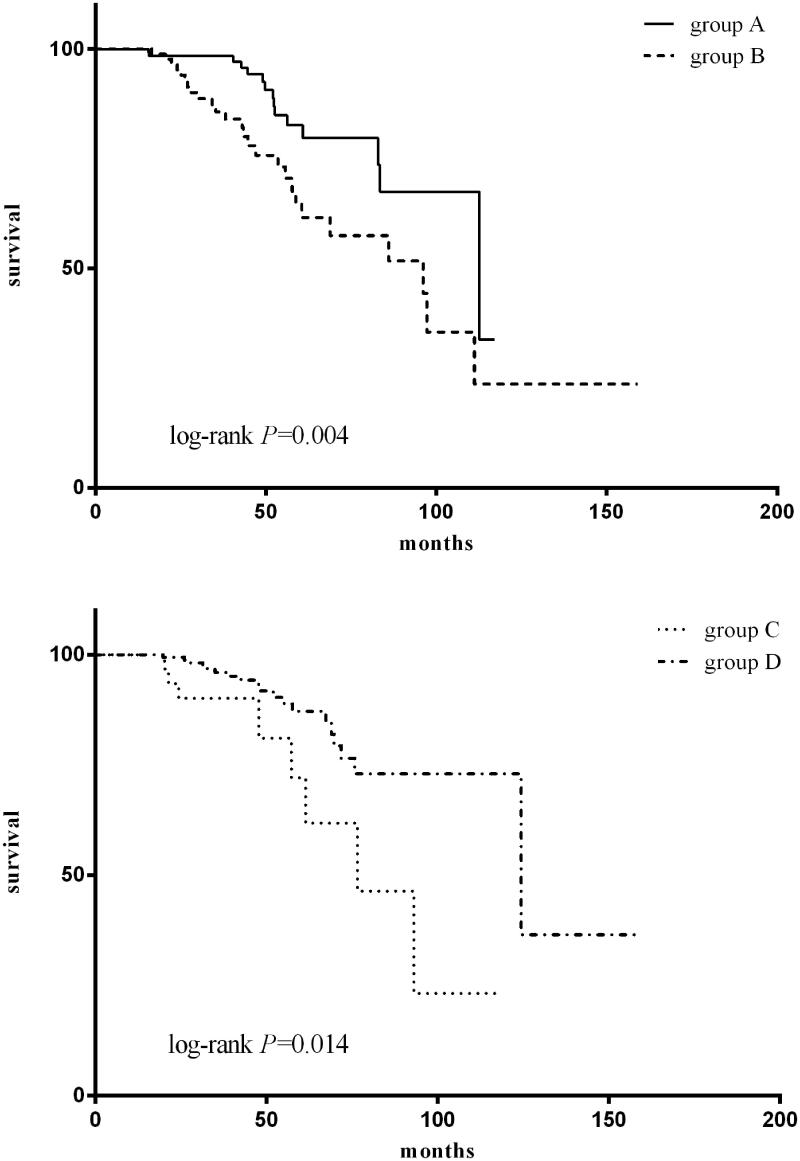
Patient survival according to serum albumin. The patients were categorized into four groups according to serum albumin at baseline and 1 year after PD: group A, serum albumin <35 g/L at baseline as well as 1 year after PD; group B, serum albumin <35 g/L at baseline and ≥35 g/L at 1 year after PD; group C, serum albumin ≥ 35g/L at baseline and <35 g/L at 1 year after PD; group D, serum albumin ≥ 35g/L at baseline as well as 1 year after PD.

The patients were categorized into four groups according to serum albumin at baseline and 1 year after PD: group A, serum albumin <35 g/L at baseline as well as 1 year after PD; group B, serum albumin <35 g/L at baseline and ≥35 g/L at 1 year after PD; group C, serum albumin ≥ 35g/L at baseline and <35 g/L at 1 year after PD; group D, serum albumin ≥ 35g/L at baseline as well as 1 year after PD. The results show that for the patient whose albumin levels <35 g/L at baseline (groups A and B), the group B patients had better outcomes compared to group A (Figure 3), with 5 years survival rates of 82.6% versus 64.6%. For the patients whose serum albumin ≥35 g/L at baseline (groups C and D), the group C patients had poorer survival compared to group D (Figure 3), with 5 years survival rates of 72.1% versus 87.2%. Since univariate analysis showed that age, baseline urine volume, as well as albumin, pre-albumin, parathyroid hormone, and C-reaction protein at 1 year of peritoneal dialysis were associated with patient survival, these factors were added to the multivariate Cox regression analysis. In this model, age and albumin at 1 year after peritoneal dialysis were independent predictors of all-cause mortality (Table 4). Every 1 year of age rise would result in a 3.9% increase in the risk of mortality (HR = 1.039, 95%CI 1.016–1.061, *p* = 0.001). Every 1 g/L increase in albumin at 1 year after PD confers an 8.7% decrease in the risk of mortality (HR = 0.913, 95%CI 0.856 –0.973, *p* = 0.005).

## Discussion/conclusion

Serum albumin serves as an indicator of nutritional status in patients with end-stage kidney disease [8]. Growing evidence has proven that hypoalbuminemia at baseline as well as baseline high serum C-reactive protein to albumin ratio [14] or platelet-to-albumin ratio [15], the biomarkers to monitor inflammation and nutrition, are important predictors of mortality in peritoneal dialysis. However, the levels of serum albumin are dynamically changed during PD. In the past, it was reported that serum albumin achieved the peak levels after 1 year of PD, regardless of the initial albumin levels [13]. In our study, we found that the serum albumin was increased at 1 year after PD compared to baseline, although the protein loss from PD effluent was increased after 1 year of PD. The reasons for this appearance were not clear. Nutritional intervention, as well as improvement of gastrointestinal symptoms after removal of uremic toxins, may be involved in the improvement of hypoproteinemia after PD.

It was reported that the initial serum albumin or single point serum albumin level, such as at the beginning, steady-state, peak-stage, at the end of the PD, were significantly associated with mortality [1,12,13]. In addition, averaged serum albumin was also found to be associated with mortality [16]. In the present study, we wonder whether the changes of serum albumin during PD influence mortality. We found that the survival rates were increased by improvement of albumin even if with a lower initial serum albumin level (groups A and B). While in the groups with a higher initial serum albumin level (groups C and D), the survival rates were decreased along with the decrement of albumin after peritoneal dialysis. Furthermore, there were no differences in mortality between groups A and C. We further analyzed sex, age, BMI, primary renal disease, as well as urine volume, hemoglobin, eGFR, blood pressure, PTH, albumin at baseline. There was no difference in these factors between the two groups except albumin at baseline, which was the same as Cox regression analysis that albumin at 1 year after peritoneal dialysis was associated with patient survival, suggesting us to pay more attention to nutrition intervention and improvement of hypoproteinemia during peritoneal dialysis.

Although peritoneal dialysis is becoming more widespread, PD among diabetic patients carries some concerns, such as massive protein leakage from dialysate effluent aggravated hypoproteinemia and edema. In our study, the serum albumin levels were low in the 79 diabetes patients at baseline (data not shown). After PD, although protein leakage from peritoneal dialysate and urine [8], the levels of albumin were increased, and the change of albumin during the first year of PD was the same as non-diabetes patients. In addition, multivariate Cox regression analysis show that diabetes did not affect mortality, indicating that diabetes patients can also derive benefit from PD as well as non-diabetes patients in improving hypoproteinemia as well as reducing mortality.

There were some limitations in the study. First, it was a single-center retrospective study and the sample size was relatively small, which requires further research using a multi-center prospective cohort design to verify the results. Second, our study did not include fluid volume status as well as nutritional status, which were thought to be influenced serum albumin, therefore needs to be added further research.

In conclusion, from this cohort observation study, we found that level of serum albumin was increased in the first year of PD. Serum albumin after 1 year of PD predicted mortality in peritoneal dialysis, suggesting we pay more attention to timely nutrition intervention and improvement of hypoproteinemia during the first year of peritoneal dialysis.
